# What Role Does the Prefrontal Cortex Play in the Processing of Negative and Positive Stimuli in Adolescent Depression?

**DOI:** 10.3390/brainsci9050104

**Published:** 2019-05-07

**Authors:** Siyabend Kaya, Ciara McCabe

**Affiliations:** School of Psychology and Clinical Language Sciences, University of Reading, Reading RG6 6AL, UK; m.s.kaya@pgr.reading.ac.uk

**Keywords:** depression, adolescent, prefrontal cortex, neural, reward, positive and negative

## Abstract

This perspective describes the contribution of the prefrontal cortex to the symptoms of depression in adolescents and specifically the processing of positive and negative information. We also discuss how the prefrontal cortex (PFC) activity and connectivity during tasks and at rest might be a biomarker for risk for depression onset in adolescents. We include some of our recent work examining not only the anticipation and consummation of positive and negative stimuli, but also effort to gain positive and avoid negative stimuli in adolescents with depression. We find, using region of interest analyses, that the PFC is blunted in those with depression compared to controls across the different phases but in a larger sample the PFC is blunted in the anticipatory phase of the study only. Taken together, in adolescents with depression there is evidence for dysfunctional PFC activity across different studies and tasks. However, the data are limited with small sample sizes and inconsistent findings. Larger longitudinal studies with more detailed assessments of symptoms across the spectrum are needed to further evaluate the role of the PFC in adolescent depression.

## 1. Depression a Global Burden

Depression is currently reported as the most important cause of worldwide ill health. In the last 10 years, the depression rate has increased by more than 18% [1] and more than 300 million people worldwide now suffer from depression [1]. Unfortunately, a completely effective treatment has not been developed for individuals suffering from depression. Although current pharmacotherapy and psychotherapy helps many patients, they have limited efficacy and significant adverse effects [2].

Depression differs from the emotional fluctuations shown in daily life events, and long-term depression, in particular, can lead to serious health problems. It can prevent an individual from fulfilling his/her potential in school, in society, in family, and in work life. There are also high rates of depression in those that commit suicide and approximately 800,000 people die from suicide each year, with suicide being the second leading cause of death among 15–29-year-olds [1].

## 2. Adolescent Depression

Adolescent depression is defined by the diagnostic statistical manual (DSM) as having five or more symptoms present during a two week period; (1) depressed or irritable, cranky mood (outside being frustrated) or (2) loss of interest or pleasure and any three of the following: Significant weight loss or decrease in appetite (more than 5% of body weight in a month) or failure to meet expected weight gains, insomnia or hypersomnia, psychomotor agitation or retardation, fatigue or lack of energy, feelings of worthlessness or guilt, decreased concentration or indecisiveness, or recurrent thoughts of death or suicide [3].

Early onset depression, in particular during the adolescence period, has a destructive effect that predicts worse health outcomes in later life [2]. Therefore, understanding the underlying mechanisms of depression in adolescence is needed if we are to improve treatment and initiate prevention [4]. Adolescents experience dynamic changes in their social relations, thus leading to a very complex emotional life, while at the same time deal with hormonal and neural changes [5]. As adolescence is a time of fluctuating positive and negative emotional experiences, it is a vulnerable time for depression. However, as with adult depression described above, there is a heterogeneity in symptoms of adolescent depression and it is not entirely clear how they map to neurobiology [6]. One reason for this has been inconsistent use of questionnaires and assessments in research, which lack any specific detail about mood change in depression [7]. Understanding adolescent depression could benefit from more detailed measures of specific symptoms, e.g., anhedonia (the reduced experience of interest and pleasure), which is currently usually only assessed via its presence or absence and not in any detail from the adolescent experience [7]. In an attempt to do this, we recently collected information on the adolescent experience of depression and anhedonia through qualitative interviews and found that a number of adolescents described a blunting of all emotion (positive and negative) and not just positive emotion, which may have been expected [8]. However, this fits with the view of a recent meta-analysis also suggesting overall blunting in positive and negative emotions in depression [9]. Still yet, more needs to be done to illuminate the adolescent depression experience perhaps using experiential sampling methodology, which is the changes in symptoms during daily life, if we are to develop even more efficacious personalised treatments.

Recent methodological developments have led to significant improvements in understanding the neurobiological mechanisms of the emotional lives of adolescents [10,11]. Owing to these improvements, it is reported that in the course of adolescence, neural networks are reconstructed with both increases and decreases in white and grey matter, respectively [12,13]. These changes have been suggested as underlying age differences in behaviour for e.g., it is thought that adolescents are more sensitive to peer relationships than adults because they have more activation in areas involved in socialisation, such as the medial prefrontal cortex (mPFC), compared to adults [12]. 

## 3. Neurobiology of Depression

At the biological level, depression can have wide spread effects on the brain [14]. Studies old [15,16,17] and new [18,19,20] find that depression affects many brain regions. Prominent brain regions with both functional and structural differences in depression are the frontolimbic brain regions such as the amygdala, hypothalamus, and prefrontal cortex (PFC) [21,22] (for review, see References [23,24]). Dysfunctional connectivity between these regions via an amygdala–striatal–pallidial–thalamic–cingulate cortex circuit is also found dysfunctional in depression [14,25]. Interestingly, a recent review of voxel-level resting state functional connectivity (RSFC) suggests that the lateral orbitofrontal cortex, which projects to the ACC, has increased sensitivity to non-rewards in depression whereas the more medial orbitofrontal cortex reward system is underactive in depression [26]. Moreover, Rolls et al. in 2018 found that unmedicated patients with depression primarily had increased RSFC between the subcallosal anterior cingulate with the lateral orbitofrontal cortex, between the pregenual/supracallosal anterior cingulate and the medial orbitofrontal cortex, and between parts of the anterior cingulate with the inferior frontal gyrus, superior parietal lobule, and with early cortical visual areas. Further, this study reported that the RSFC was reduced in depressed patients that were medicated [26]. Interestingly, a recent study has also found that increased pretreatment pregenual anterior cingulate cortex activity to sad vs. happy faces was observed in responders relative to nonresponders, and that anterior cingulate cortex activity was able to predict response status at the level of the individual participant [27]. Given the importance of such networks of activity in the pathophysiology of depression and its treatment, how they might also act as predictors for adolescent depression could aid the identification of new targets for intervention approaches. In this respect, studies have emphasized that the PFC is one of the most important cortical brain regions in a network of regions in depression and is therefore a possible target for treatment and prevention in adolescents [28]. 

## 4. The Importance of the PFC

The role of the PFC in behaviours involving cognitive control and emotion processing has been well documented in adults (for review, see Reference [29]) and adolescents [30]. In summary, the dlPFC is mostly associated with cognitive processes such as target-oriented behaviours and attention control [31,32] while the vlPFC has a significant role in complex processes such as the self-regulation of emotion [19,33].The vmPFC, on the other hand, has been shown to play a role in the production of negative emotions [34,35,36,37].

Interestingly, during adolescence, the structural maturation of the PFC is suggested to underlie the maturation of emotion regulation strategies. Studies consistently find normative thinning of the grey matter during adolescence which has been identified as an adaptive process in longitudinal studies on cognition [38]. For example, Shaw et al. [39] found that adolescents that exhibited greater peak thickness around puberty, followed by greater cortical thinning into adulthood had superior intellectual abilities. Furthermore, greater cortical thinning of the left dlPFC and left vlPFC during adolescence has been found to predict greater use of cognitive reappraisal, the ability to negotiate emotionally stressful situations by being more optimistic, reinterpreting the stressful stimuli, and actively mending their negative mood, in healthy females [38]. These findings suggest that cortical maturation may play a role in the development of adaptive emotion regulation strategies during adolescence. Interestingly, dysfunction, by way of decreased perfusion in the PFC, has been reported in patients who attempted suicide. It is thought this PFC dysfunction might reduce problem-solving ability, increase negative emotions, and, finally, aid suicidal behaviour especially given the role of the orbitofrontal cortex in response inhibition [22,40]. 

## 5. The Role of the PFC in Depression and the Processing of Negative Stimuli

The mechanisms underpinning the processing of negative emotions have received attention, as low mood and negative thinking are thought to be maintaining characteristics of depression [41]. Negative emotional stimuli activate a broad network of brain regions, including the medial prefrontal (mPFC) and anterior cingulate (ACC) cortices and, although early reviews suggested a dorsal-caudal cognitive and ventral-rostral affective subdivision [42], more recent work suggests both subdivisions make key contributions to emotional processing. Specifically, dorsal-caudal regions of the ACC and mPFC are thought to be involved in appraisal and expression of negative emotion, whereas ventral-rostral portions of the ACC and mPFC seem to have a regulatory role with respect to limbic regions involved in generating emotional responses [43]. Examining these systems in relation to low mood, a study by Aoki and colleagues [44] using the neuroimaging tool optical topography, found that adults experiencing higher levels of negative moods showed lower levels of PFC activity during a verbal working memory task. This also replicated the results of their previous study based on an independent sample [45]. In another study using near infrared spectroscopy (NIRS), participants were asked to remember parts of their lives related to positive (happiness) and negative (anger) feelings, and at the same time, heart rate changes were measured. The authors found that changes in oxyhemoglobin in the bilateral PFC during silent recall of negative episodes were significantly larger than those during silent recall of positive episodes. The authors concluded that their results were important in showing that the PFC plays a key role in the cognitive control of particularly negative emotions [18]. 

As mentioned above, the dlPFC is thought to be involved in executive function and cognitive control over behaviour and action [46]. It has been found, using EEG, to have functional and structural asymmetry that correlates with depressive symptoms in healthy young adults, individuals with subclinical depression, and patients with depression [47]. Previous studies report that the left dlPFC is hypoactive for positive and the right dlPFC hyperactive for negative stimuli in depression [48] and a study by Siegle et al. [49] detected that depressed participants showed reduced dlPFC activity to negative words. Furthermore, the lateral orbitofrontal cortex (lOFC), which connects to the dlPFC, has been found to have a critical role in reversal learning and adapting behaviour based on the most positive outcome [50]. Recently, it has been posited that dysfunction of the lOFC “non-reward” circuit may lead to the generation of negative self-thoughts and reduced self-esteem apparent in depression [50,51]. 

## 6. PFC Markers of Risk for Depression and Early Life Stress (ELS)

To date, it is difficult to ascertain neural markers of risk for depression, e.g., it is not known which of the functional neuroanatomical differences seen in depressed patients predate and predict depression onset [52,53]. By examining adolescents, neurobiological studies can begin to address this issue, and studies have found heightened activity in the amygdala during facial-emotion recognition tasks [54] and greater connectivity between the amygdala and part of the PFC the subgenual anterior cingulate cortex [55]. Further, recent studies examining PFC connectivity and risk of depression by virtue of early life stress (ELS) find that adolescent females exhibited a positive association between ELS and ventrolateral prefrontal cortex (vlPFC) during implicit emotion regulation and both males and females exhibited an association between ELS and increased negative connectivity between right vlPFC and bilateral amygdala [56]. The authors suggest these results might reflect greater vlPFC activation in an emotion regulation context in response to stress i.e., under the stress accelerated hypothesis. This is where accelerated development of neural circuitry involved in emotion functioning is caused by stress and is adaptive in the short term [57]. Further, there is evidence in animal models of ELS-induced changes in mPFC function and developmental trajectory, which may be responsible for the emergence of both early-onset (during childhood and adolescence) and adulthood-onset anxiety and mood disorders [58,59]. In summary, studies find ELS, a risk factor for depression, affects PFC function in early puberty, thus indicating the importance of this region as a potential target for early intervention in those at risk. 

## 7. PFC and Resting State Functional Connectivity (RSFC) in Adolescents with Depression

Studies have begun to examine RSFC in adolescents with depression and a recent study found that depressed adolescents showed significantly greater RSFC to left amygdala, bilateral supragenual ACC, but not with PFC. The results partially support the putative dual-system hypothesis believed to underlie disorders such as major depression i.e., an imbalance between “hot” limbic activity and “cold” PFC activity. The authors suggested that adolescents have aberrant, bottom-up processing in hot limbic regions without the concomitant differences in cognitive control in cold prefrontal regions, unlike in adults with depression. In addition, changes in functional connectivity were significantly associated with changes in symptom severity after cognitive behavioural therapy. This indicates that symptom recovery may be at least partially associated with normalization of RSFC in hot emotional brain systems, and their restoration is critical for successful therapeutic interventions [60]. We have also examined resting state functional connectivity (RSFC) in adolescents and the relationship between PFC connectivity and depression symptoms. We found decreased RSFC between the amygdala and the pgACC and hippocampus and precuneus in young people with depression symptoms. We also found decreased RSFC in the young people with depression symptoms between the pgACC and the putamen and between the dmPFC and the precuneus [61]. Further, the pgACC RSFC with the insula/orbitofrontal cortex correlated inversely with the anticipation of pleasure in all subjects. Increased RSFC was observed between the pgACC and the prefrontal cortex and the amygdala and the temporal pole in the young people with depression symptoms compared to those with no symptoms. As increased connectivity between the pgACC and the insula correlated with decreased ability to anticipate pleasure, we suggest this might be a mechanism underlying the risk of experiencing anhedonia, a suggested biomarker for depression [61]. In our more recent work, we also found that in a large sample of young people with a range of depression scores, both anhedonia and depression severity related to decreased dmPFC RSFC with the precuneus, a part of the default mode network. However, we also found that increased dmPFC connectivity with the ACC/paracingulate gyrus related to anhedonia whereas increased RSFC with the frontal pole related to depression severity. This study is important as it shows us how we can dissociate symptoms in adolescents based on PFC RSFC [62]. In adolescents with depression, medial prefrontal cortical connectivity with brain regions involved in executive functioning, emotion regulation, and attention have been reported altered [28]. 

## 8. The Role of the PFC in Depression and the Processing of Positive Stimuli

In regard to positive processing specifically, studies have shown blunted neural responses that relate to positive affect [63] and depression symptoms in adolescents [64,65] and even young children [66]. Further, in relation to positive stimuli, studies of adolescents with depression report mostly decreased responses to monetary reward in regions like the ventral striatum, caudate, the dorsolateral and medial prefrontal cortex (PFC), orbitofrontal cortex (OFC), anterior cingulate cortex (ACC), and amygdala [67,68]. However, most neurobiological tasks of positive emotion processing (reward) do not examine the different phases of processing such as the anticipatory, motivational, and consummatory aspects of reward. This has led to inconsistencies across studies on reward in depression [69]. 

We have been interested in examining how young people at risk of depression respond to positive and negative stimuli both behaviourally and at the neural level. Furthermore, as recent behavioural data find that depressed adults have reduced effort expenditure for reward compared to healthy controls [70], we are also interested in how this is represented at the neural level. Therefore, to address this, we have developed an experimental model that examines the anticipation of a food reward and a consummatory phase where rewarding food is eaten. We have shown previously that those at risk of depression have decreased responses to anticipation and consummation (sight and taste of chocolate reward) in both ventral striatum and anterior cingulate cortex (ACC) [71]. We also showed that young people (16–21 years) with a family history of depression but no personal experience of depression had diminished neural responses in the orbitofrontal cortex (OFC) and the dorsal anterior cingulate cortex (dACC) to rewarding stimuli, sight, and taste combined in the at-risk group [72]. More recently, we have also shown that when examining neural activity between young people with depression symptoms and controls, using a region of interest analysis, regions like the pregenual ACC and ventral medial PFC were blunted across all positive and negative phases in adolescents with depression symptoms. We also found that whole brain analysis revealed further blunted activity in the precuneus and inferior frontal gyrus (during aversive anticipation) and hippocampus (during effort for reward) and ACC/frontal pole (during aversive consummation) in young people with depression symptoms. Further, we found a negative correlation between pgACC activity during reward consummation and anhedonia in adolescents with depression symptoms [73]. Although this was a comparatively small study, the results are in keeping with the meta-analysis and first quantitative review of emotional reactivity in depression that found consistent reductions in both positive AND negative reactivity [9] which supported our previous study [71]). This also fits with the recent hypothesis that as connectivity of the ACC, a hub for integrating cognitive, affective, and social information to guide self-regulation across domains, supports adaptive development of self-regulation during adolescence, disrupted maturation of ACC connectivity could contribute to the development of depression [74].

In our follow-up, much larger study currently under review, we found participants with depression symptoms invested less physical effort to gain the positive rewarding stimulus than controls and had blunted neural anticipation of positive and negative stimuli in the precuneus, insula, and PFC (left dlPFC and lOFC) and blunted neural effort for positive in the putamen [75]. As the dlPFC is involved in cognitive control and in executive functions [47], we suggest dysfunction in this region might indicate a mechanism by which reduced planning to gain positive and avoid negative stimuli might arise in those with depression symptoms. As the lOFC connects to the dlPFC, insula, and premotor areas [76] and has been found to have a critical role in reversal learning and adapting behaviour based on the most rewarding outcome [76], reduced lOFC activity might disrupt ability to switch behaviour, which, in those depressed, might affect preparation to gain reward or avoid aversion. 

## 9. Conclusions

Taken together, the literature on the neural activity related to positive and negative emotion processing is limited in adolescent psychopathology [7,64]. Depression, notwithstanding decades of studies, is a serious disorder with early onset in adolescence indicating worse long-term outcomes, yet the neural basis of adolescent depression is not yet fully understood [7]. Although the PFC is a key region implicated in the processing of positive and negative emotion, some inconsistencies in direction of effects are present in the literature [23,77]. Therefore, in order to understand the role of PFC in adolescent depression, further studies are needed that examine the processing of positive and negative stimuli in a dimensional fashion across the spectrum, in line with an RDoC type approach. Further, it would also be of interest to examine how adolescents regulate their emotion processing in relation to PFC activity over time and depression onset [38]. Adolescence is an important time, in which both physical and mental changes are experienced. Although studies have shown that the PFC region is implicated in depression in adults, less is known about how the PFC impacts upon negative and positive moods in the adolescent period. Knowing how the PFC is involved in major symptoms like anhedonia could allow us to develop more targeted interventions for youth depression. 
